# Partial Hepatectomy and Ablation for Survival of Early-Stage Hepatocellular Carcinoma Patients: A Bayesian Emulation Analysis

**DOI:** 10.3390/life14060661

**Published:** 2024-05-22

**Authors:** Jiping Wang, Yunju Im, Rong Wang, Shuangge Ma

**Affiliations:** 1 Department of Biostatistics, Yale School of Public Health, New Haven, CT 06510, USA; shuangge.ma@yale.edu; 2 Department of Biostatistics, University of Nebraska Medical Center (UNMC), Omaha, NE 68198, USA; yim@unmc.edu; 3 Department of Chronic Disease Epidemiology, Yale School of Public Health, New Haven, CT 06510, USA; r.wang@yale.edu; 4 Yale Cancer Outcomes, Public Policy and Effectiveness Research (COPPER) Center, New Haven, CT 06520, USA

**Keywords:** comparative effectiveness, HCC, target trial emulation, Bayesian survival, SEER-Medicare

## Abstract

Partial hepatectomy and ablation therapy are two widely used surgical procedures for localized early-stage hepatocellular carcinoma (HCC) patients. This article aimed to evaluate their relative effectiveness in terms of overall survival. An emulation analysis approach was first developed based on the Bayesian technique. We estimated propensity scores via Bayesian logistic regression and adopted a weighted Bayesian Weibull accelerated failure time (AFT) model incorporating prior information contained in the published literature. With the Surveillance, Epidemiology, and End Results (SEER)-Medicare data, an emulated target trial with rigorously defined inclusion/exclusion criteria and treatment regimens for early-stage HCC patients over 66 years old was developed. For the main cohort with tumor size less than or equal to 5 cm, a total of 1146 patients were enrolled in the emulated trial, with 301 and 845 in the partial hepatectomy and ablation arms, respectively. The analysis suggested ablation to be significantly associated with inferior overall survival (hazard ratio [HR] = 1.35; 95% credible interval [CrI]: 1.14, 1.60). For the subgroup with tumor size less than or equal to 3 cm, there was no significant difference in overall survival between the two arms (HR = 1.15; 95% CrI: 0.88, 1.52). Overall, the comparative treatment effect of ablation and partial hepatectomy on survival remains inconclusive. This finding may provide further insight into HCC clinical treatment.

## 1. Introduction

Hepatocellular carcinoma (HCC) accounts for more than 90% of primary liver cancers [1] and is the fourth leading cause of cancer-related death globally [2]. The risk of developing HCC increases with age [3,4,5]. In Europe and North America, the average age of HCC diagnosis ranges from around 63 to 65 years and up to 75 years in some populations [6]. In Japan, the age distribution of patients with HCC peaks at nearly 70 years, and this number is still increasing [7]. HCC is considered a life-limiting factor even in very old patients [8]. Comparatively, the clinical management of elderly HCC patients is more complicated because of the higher prevalence of comorbidities including cardiovascular diseases, respiratory diseases, and diabetes mellitus [5]. There is a strong and rising demand for research on the treatment and outcomes of older HCC patients [5,7].

Effectively treating early-stage HCC for the elderly remains a challenging clinical question. For early-stage localized HCC, partial hepatectomy and ablation therapy are the most commonly used treatment options in clinical practice without donor constraints [9] and can achieve acceptable survival outcomes [10,11,12]. Partial hepatectomy or liver resection, defined as the surgery to remove the part of the liver where cancer is found, includes removing a wedge of tissue, an entire lobe, or a larger part of the liver, along with some of the healthy tissue around it. Ablation therapy removes or destroys tissue and includes radiofrequency, microwave, cryoablation, and electroporation therapy. Partial hepatectomy is widely accepted as a curative treatment for older patients. A report showed that patients over 70 years of age constituted more than 50% of those undergoing hepatectomy for HCC [13]. However, this procedure may negatively impact the already compromised function of livers with chronic conditions such as cirrhosis [14]. Compared to partial hepatectomy, liver ablation may be more conservative, safe, and effective for older patients [15], but it is more likely to result in residual tumors, and technical feasibility may be suboptimal [16]. The choice between partial hepatectomy and ablation for HCC is still being widely debated. However, there has been a lack of randomized clinical trial (RCT) or causal inference analysis comparing the effectiveness of the two procedures among older patients.

Among the available observational data analysis techniques that may draw randomized clinical trial (RCT)-type conclusions, emulation has emerged as a strong option, given its trial-like architecture, interpretability, and scalability [17,18,19]. The emulation technique has been well developed through a long list of publications and has also been successfully applied to evaluate treatment effectiveness among cancer patients [17,18,19,20,21,22,23,24]. The dominating majority of the existing emulation studies belong to the frequentist domain, and estimation and inference are solely based on the data in hand. As shown below, for our research question, even with the large SEER-Medicare data, the sample size is still somewhat limited. Additionally, there are quite a few relevant published studies examining related questions. As such, it is natural to resort to the Bayesian paradigm, which offers a powerful framework for estimating treatment effects and can “combine” prior knowledge with observed data. The Bayesian paradigm is also able to accommodate small sample sizes and yield more reliable and informative estimates. There are multiple ways of generating prior information, and the summarized information from published studies can serve as a reliable source.

We note that for real late-phase RCTs (that can potentially be sufficiently powered), applications of Bayesian techniques have been limited [25,26]. This is because, for many real RCTs (for example, those focused on new drugs), there is usually a lack of directly relevant prior information. We note that this is quite different from our emulation analysis of two already commonly adopted and extensively investigated surgical procedures. Additionally, there is a risk that the analysis may be negatively impacted by the “subjectiveness” in specifying prior distributions. We note that this can be tackled by specifying fewer informative priors as done in this study. Emulation analysis, despite its strong assembly of RCT analysis, is still an observational data analysis technique, and there have been many Bayesian methods and analyses of observational data [27,28]. Our literature review also suggests that there has been some, albeit limited, development of Bayesian emulation analysis [29,30]. However, the existing development has not delivered a full analysis pipeline or taken full advantage of state-of-the-art Bayesian techniques.

This study has two main goals. The first is to evaluate the comparative effectiveness of partial hepatectomy and ablation therapy on overall survival among older patients with early-stage HCC, with tumor size less than or equal to 5 cm (or 3 cm). To the best of our knowledge, this is the first head-to-head comparison in an RCT-like context, and the finding can be informative for HCC clinical treatment. The second goal is to develop the Bayesian emulation analysis pipeline and approach, which can enjoy multiple advantages of Bayesian analysis and provide a useful alternative to its frequentist counterpart. This new analysis approach can be potentially applied to other diseases, clinical outcomes, and treatments.

## 2. Materials and Methods

The SEER-Medicare data were utilized in this study. SEER (Surveillance, Epidemiology, and End Results) contains 22 cancer registries in the United States (U.S.). Medicare is a federally funded health insurance program in the U.S. In 2010, approximately 94% of the U.S. population aged 65 years or older was enrolled in Medicare [31]. The SEER-Medicare data provide an ideal opportunity to study the U.S. cancer population over 65 years old and has served as the basis of many analyses including emulation analysis [23,24]. Data analyzed in this study were obtained through a data use agreement by the NCI.

**Figure 1:**
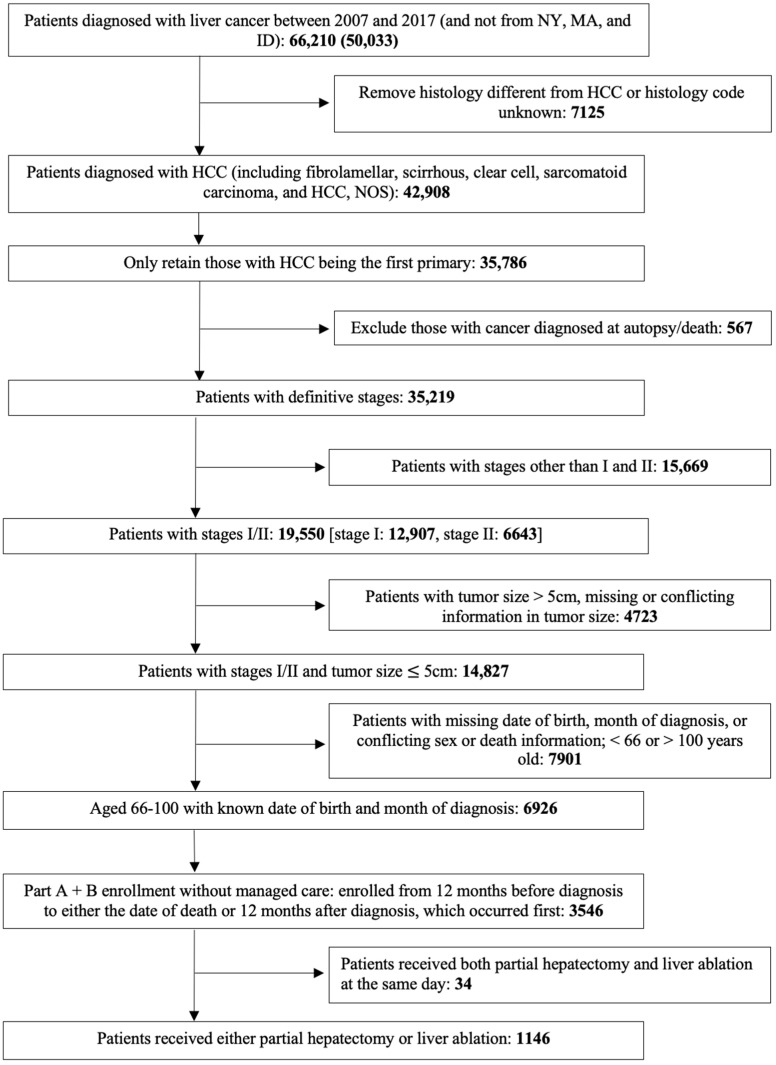
Flowchart for sample selection.

The design was strongly motivated by relevant trials [11,16,32] and observational analyses [9,33,34], while taking into consideration data availability in the SEER-Medicare database. The flowchart of emulated trial patient selection for the main cohort is summarized in Figure 1, and more detailed information was provided in Supplementary Materials. Briefly, the emulated trial enrolled Medicare beneficiaries with first primary HCC who were (1) 66–100 years old at diagnosis; (2) diagnosed between 1 January 2007 and 31 December 2017; (3) stages I–II; (4) tumor size ≤ 5 cm; (5) received partial hepatectomy or liver ablation (including all types of ablation) within one year after diagnosis; and (6) had continuous enrollment in Medicare Part A and Part B, with no health maintenance organization (HMO) enrollment from one year before to one year after cancer diagnosis or death (whichever came first). A patient was excluded if he/she (1) was reported from the registries in New York (NY), Massachusetts (MA), and Idaho (ID) because of missing cancer-related information; (2) had missing information on the month of diagnosis, stage, or tumor size; (3) was reported from death certificate or autopsy; and (4) received both procedures on the same day. Beyond the main cohort, we also conducted a subgroup analysis on patients with tumor sizes less than or equal to 3 cm, as some studies suggested that ablation may be preferable for this group [35]. All other eligibility criteria remained unchanged.

By reviewing relevant published studies, and also taking data availability into consideration, we included the following variables as potential confounders, which may have an impact on treatment initiation (choice of surgeries) and the outcome (overall survival): (1) baseline demographics: age, sex, race, and marital status; (2) cancer-related variables: tumor size and stage; (3) comorbidities: Elixhauser comorbidity scores [36]; and (4) liver diseases: viral and nonviral hepatitis, alcoholic liver disease, non-alcoholic cirrhosis, portal hypertension, and hepatic encephalopathy or coma within one year before the first HCC diagnosis. For the main cohort, marital status was observed to be balanced in the two groups and was treated as a covariate and only included in the outcome model to improve precision. All the other confounders were included in both the propensity score model and the outcome model. The treatment group was defined as partial hepatectomy (ablation) if a patient received partial hepatectomy (ablation) as the first surgery procedure after diagnosis; this was done to estimate an intention-to-treat effect.

Our outcome of interest was overall survival. The patient’s vital information was obtained from the Medicare records. Time zero was set as the time of surgery. Immortal time bias, if any, was expected to be small, as the two treatment groups received comparable treatments and the waiting time was relatively short. For patients who underwent multiple ablation sessions, time zero was set as the time of the first ablation. For patients (*n* < 11) who received both procedures during the first year after diagnosis, we censored them at the time of receiving the second type of surgery. All subjects were followed to the time of receiving another procedure of interest, the end of 2019, or death (whichever happened first).

In Bayesian analysis, prior information is incorporated to assist estimation of the treatment effect. To obtain prior information, we searched PubMed using the keywords “(hepatocellular carcinoma) AND (resection) AND ((ablative) OR (ablation))”. To obtain the most updated information, we limited our search to articles published between June 2020 and June 2022. Our preliminary exploration suggested that publications on treatment assignments in clinical practice were very limited. As such, the literature review and prior information extraction were focused on overall survival. With the resulting papers, we manually checked titles and abstracts to identify those that involved evaluating the treatment effects of resection and ablation. Further, we reviewed each paper to identify those that reported survival analysis results. About 300 papers emerged in our preliminary search. Among them, our review suggested that 17 evaluated the treatment effect of interest. The list of these 17 papers is provided in Section S2 of the Supplementary Materials, where we also provided information on their main findings (including estimated HR (hazard ratio), 95% CI (confidence interval), and *p*-value). Their median HR was calculated as 1.631, with a median absolute deviation (MAD) of 0.497. The prior of the treatment effect was specified based on these estimates and incorporated into the estimation. It is expected that, as in other Bayesian analyses, this can facilitate more accurate and robust inference regarding the treatment effect on overall survival.

It is noted that Bayesian analysis does not demand prior information to be “fully correct”. As such, our literature review and information extraction do not need to be as stringent as in, for example, a meta-analysis. In analysis, the examination of the trace plots suggested satisfactory convergence and sufficient iterations. Additionally, sensitivity analysis suggested that the analysis results were not sensitive to the prior specifications. Details are available from the authors.

For the statistical methods, first, a Bayesian logistic regression [37,38] was conducted using R package MCMCpack version 1.7-0 [39]. The baseline confounders described above were included as covariates. As there was no prior information on treatment assignment, a diffuse normal prior, which has marginal means = 0, marginal variances = 100, and covariances = 0, was imposed. A total of 10,000 MCMC (Markov chain Monte Carlo) iterations were performed for inference, with the first 5000 as the burn-in period. With the 5000 sets of obtained propensity scores, thinning was applied to the Markov chain, and 500 sets of propensity scores were selected based on every 10th iteration. It is noted that these settings/operations are standard in the Bayesian literature. Then, the weight was calculated as the inverse of the propensity score for one treatment group and the inverse of one minus propensity score for the other group. Stabilized weights with a truncation at the upper 99.5% percentile (that is, if a weight was over the 99.5% percentile, it was set as the 99.5% percentile) were adopted for retaining the same group size ratio and avoiding putting extreme weights on certain subjects. By assigning each set of weights to the study subjects, we created a pseudo-population, for which there existed no association between the baseline confounders and received treatment. Here we note that, different from the frequentist analysis, multiple sets of weights and multiple pseudo-populations were created, accommodating the uncertainty associated with estimating the propensity scores.

In the second step, the Bayesian Weibull AFT (accelerated failure time) model was fitted to derive the treatment effect using the weighted pseudo-populations [40]. We assigned a weakly informative normal prior to the log hazard ratio of the treatment effect, where the parameters were set based on the prior information mining. For each of the baseline covariates, we assigned a normal prior with a mean of 0 and a standard deviation of 10, which is sufficiently non-informative. For the shape parameter of the Weibull distribution, we assigned an exponential prior with a rate of 1. These settings are popular in Bayesian survival analysis. In estimating the outcome model, for each weighted pseudo-population, we run an MCMC sampler with 20,000 iterations, with the first 10,000 as the burn-in period. Inference was then conducted using the 500 × 10,000 MCMC samples. For each variable, our interest is its estimate and 95% credible interval.

In our analysis, the posterior distributions did not have simple analytic forms. For computation, we resorted to MCMC. To check the convergence of the MCMC algorithms, we ran five independent MCMC chains and visually inspected the trace plots. The agreement between the five chains was also examined to confirm that sufficient iterations had been run. Further, convergence was examined by the Gelman–Rubin potential scale reduction factor (PSRF) [41]. In our main analysis, for estimating the propensity scores, all parameters exhibited PSRF less than 1.02. For estimating the treatment effect, all parameters exhibited PSFR equal to 1. These statistics suggested convergence. The subgroup analysis had the same satisfactory convergence.

## 3. Results

**Figure 2:**
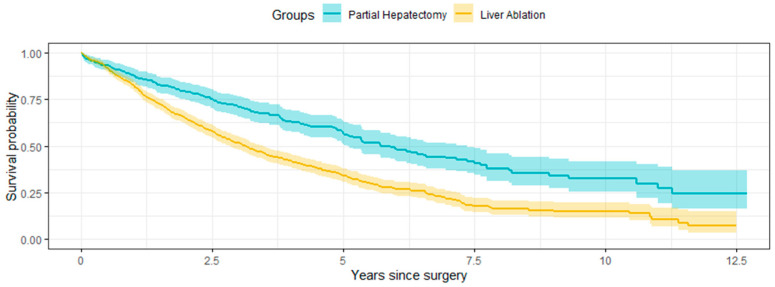
Kaplan–Meier survival curves for the unweighted population stratified by surgery for the main cohort.

The final main cohort included a total of 1146 eligible participants, with 301 in the partial hepatectomy group and 845 in the ablation group (Figure 1). The waiting time from diagnosis to surgery was observed to be relatively short, with a median of 71 days and over 90% of the main surgeries performed within 200 days after diagnosis. The immortal time bias was relatively small, and a one-year window to capture the first surgery following diagnosis was considered sufficient. As summarized in Table 1, before the IPT weighting, the participants treated with ablation were more likely to have smaller tumors and have more liver-related conditions except for hepatitis B and other viral hepatitis. The overall mortality of the partial hepatectomy group was 49.2%, and the median survival time was 5.7 years (Figure 2). For the ablation group, they were 66.4% and 3.1 years, respectively. Without adjustment for the confounders, patients who received partial hepatectomy had a longer overall survival (*p*-value < 0.001 by a log-rank test).

The Bayesian Logistic regression results are summarized in Table 2. Multiple variables were observed to have significant associations with treatment assignment, including age at diagnosis, tumor size, alcoholic liver disease, non-alcoholic cirrhosis, and portal hypertension. For the propensity score analysis, we show in Supplementary Figure S1 the distributions of the propensity scores and the corresponding weights. The distributions of the standardized mean difference (SMD) values in Supplementary Figure S2 show that most of the SMD values significantly decreased to be below or close to 0.1 with weighting, indicating the balance of the confounders. For a detailed discussion, we refer to Supplementary Section S3.

Table 3 contains the adjusted log hazard ratio and 95% credible interval for each variable using the proposed Bayesian approach. Additionally, for comparison, results from the standard frequentist approach were included in Supplementary Section S4. Overall, it was observed that the two paradigms had qualitatively similar but quantitatively different estimates. Ablation was observed to have a significant association with survival, with an estimated log HR of 0.30 (HR = 1.35) and 95% credible interval (CrI) (0.13, 0.47); the corresponding 95% credible interval for HR is (1.14, 1.60). Other variables significantly associated with survival were age at diagnosis, marital status, tumor size, Elixhauser comorbidity score, Hepatitis B, portal hypertension, and hepatic encephalopathy/coma.

### Subgroup Analysis Results

**Figure 3:**
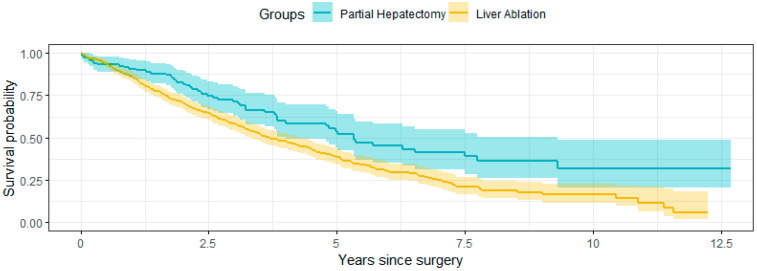
Kaplan–Meier survival curves for the unweighted population stratified by surgery for the subgroup with tumor size under 3 cm.

For the subgroup analysis with tumor size less than or equal to 3 cm, the final cohort included a total of 660 eligible participants, with 116 in the partial hepatectomy group and 544 in the ablation group. Additional information on sample selection is available from the authors. The overall mortality of the partial hepatectomy group was 46.6%, and the median survival time was 5.3 years (Figure 3). For the ablation group, they were 61.9% and 3.7 years, respectively. Similarly to the main cohort, without the adjustment for the confounders, patients who received partial hepatectomy had a longer overall survival (*p*-value < 0.001 by a log-rank test). The Bayesian Logistic regression results and the Bayesian survival analysis results were summarized in Supplementary Tables S6 and S7. We also examined the balance of the two arms after weighting and found similar performance as the main analysis. Some results different from the main analysis were obtained. Specifically, tumor size was no longer significant in choosing the surgical procedures. While ablation had an association with worse survival, this effect was no longer statistically significant, with an estimated log hazard ratio of 0.14 (hazard ratio = 1.15) and a 95% credible interval of (−0.13, 0.42); the corresponding 95% credible interval for HR is (0.88, 1.52). Other variables significantly associated with survival were age at diagnosis, marital status, Elixhauser comorbidity score, Hepatitis B, portal hypertension, and hepatic encephalopathy/coma.

## 4. Discussion

Effectively treating early-stage HCC for the elderly remains a challenging clinical question. There have been extensive debates on the relative effectiveness of different surgical options, and different studies have drawn conflicting conclusions [42]. To further elucidate this question, in this study, we resorted to real-world evidence contained in SEER-Medicare and evaluated the comparative effectiveness of partial hepatectomy and liver ablation for stage I-II HCC with tumor size less than or equal to 5 cm (or 3 cm) for patients over 66 years old. With a new Bayesian emulation analysis, we concluded that partial hepatectomy was superior in overall survival for the main cohort with tumor size under 5 cm but found no significant difference between the two procedures for the subgroup with tumor size under 3 cm. This finding is consistent with some published studies showing a superior effect of partial hepatectomy [43,44,45] or no significant difference [46,47,48] but deviates from those that concluded the superiority of ablation [49]. Such differences may arise from differences in target populations, definitions of treatments, analytical approaches, and others.

This study may have limitations. The SEER-Medicare data have broad coverage, and our findings can be reasonably extended to the whole U.S. population with the defined demographic/clinical characteristics. However, their broader generalizability should still be taken with caution. Emulation has been demonstrated as powerful. On the other hand, its limitations have also been acknowledged [17,18,19,20], including limited information, possible imbalance in variables not collected, and others.

The biggest difference in this study is the adoption of Bayesian analysis. For real RCTs, there have been more and more applications of Bayesian techniques, and this effort has support from the FDA. There are many more applications of Bayesian techniques in observational data analysis, and this study is among the first to introduce Bayesian techniques to emulation analysis. Bayesian emulation analysis accounts for the uncertainty of the estimated propensity scores and accommodates prior information. With its unique importance, our prior information mining had been focused on the treatment effect. It is possible to extend this effort to the other variables, although it is noted that this effort is not expected to change the findings significantly. The similarities and differences between the proposed Bayesian analysis and the frequentist analysis are “assuring” and also demonstrate the impact of incorporating prior information. This “replicability” does not invalidate Bayesian analysis but can instead provide support for conducting more Bayesian analysis in real and emulated clinical trial studies.

## Figures and Tables

**Table 1:** Patient demographics, cancer-related characteristics, and comorbidities for the whole main cohort and stratified by treatment.

	Partial Hepatectomy (*n* = 301)	Ablation (*n* = 845)	Total (*n* = 1146)	*p*-Value
**Demographics**				
Age at diagnosis, mean (sd)	73.5 (5.49)	73.6 (5.70)	73.6 (5.64)	0.961
Female	112 (37.2%)	319 (37.8%)	431 (37.6%)	0.922
Non-Hispanic White	148 (49.2%)	460 (54.4%)	608 (53.1%)	0.132
Married	176 (58.5%)	494 (58.5%)	670 (58.5%)	>0.999
Year of diagnosis				
2007	26 (8.6%)	46 (5.4%)	72 (6.3%)	0.389
2008	19 (6.3%)	58 (6.9%)	77 (6.7%)	
2009	15 (5.0%)	53 (6.3%)	68 (5.9%)	
2010	28 (9.3%)	59 (7.0%)	87 (7.6%)	
2011	26 (8.6%)	63 (7.5%)	89 (7.8%)	
2012	29 (9.6%)	73 (8.6%)	102 (8.9%)	
2013	28 (9.3%)	97 (11.5%)	125 (10.9%)	
2014	28 (9.3%)	67 (7.9%)	95 (8.3%)	
2015	32 (10.6%)	110 (13.0%)	142 (12.4%)	
2016	31 (10.3%)	112 (13.3%)	143 (12.5%)	
2017	39 (13.0%)	107 (12.7%)	146 (12.7%)	
**Tumor characteristics**				
Tumor size (mm)	33.5 (11.0)	27.8 (9.87)	29.3 (10.5)	<0.001
Stage II	80 (26.6%)	253 (29.9%)	333 (29.1%)	0.303
**Comorbidities**				
Elixhauser comorbidity score	22.49 (9.06)	22.62 (8.84)	22.59 (8.89)	0.833
Hepatitis B	67 (22.3%)	125 (14.8%)	192 (16.8%)	0.004
Hepatitis C	123 (40.9%)	410 (48.5%)	533 (46.5%)	0.026
Other/unspecified viral hepatitis	24 (8.0%)	60 (7.1%)	84 (7.3%)	0.711
Nonviral hepatitis	40 (13.3%)	185 (21.9%)	225 (19.6%)	0.002
Alcoholic liver disease	29 (9.6%)	192 (22.7%)	221 (19.3%)	<0.001
Nonalcoholic cirrhosis	204 (67.8%)	749 (88.6%)	953 (83.2%)	<0.001
Portal hypertension	75 (24.9%)	471 (55.7%)	546 (47.6%)	<0.001
Hepatic encephalopathy/coma	15 (5.0%)	113 (13.4%)	128 (11.2%)	<0.001

**Table 2:** Results from the Bayesian logistic regression model for estimating propensity scores.

Variable	Log Odds (95% Credible Interval)
Age at diagnosis	0.25 (0.10, 0.40)
Sex—female (ref: male)	−0.16 (−0.47, 0.16)
Race—Non-Hispanic white (ref: other)	0.00 (−0.31, 0.31)
Stage—stage II (ref: stage I)	0.19 (−0.18, 0.51)
Size (mm)	−0.52 (−0.69, −0.37)
Elixhauser comorbidity score	0.06 (−0.13, 0.22)
Hepatitis B (Yes vs. ref: No)	−0.29 (−0.66, 0.08)
Hepatitis C	0.12 (−0.18, 0.45)
Other or unspecified viral hepatitis	−0.06 (−0.57, 0.49)
Nonviral hepatitis	0.37 (−0.04, 0.84)
Alcoholic liver disease	0.61 (0.16, 1.05)
Nonalcoholic cirrhosis	0.84 (0.49, 1.19)
Portal hypertension	0.87 (0.55, 1.23)
Hepatic encephalopathy/coma	0.42 (−0.21, 1.12)

**Table 3:** Results from the IPT weighted Bayesian Weibull AFT models with covariates.

Variable	Log Hazard Ratio (95% Credible Interval)
Surgery—ablation (ref: partial hepatectomy)	0.30 (0.13, 0.47)
Age at diagnosis	0.24 (0.16, 0.31)
Sex—female (ref: male)	−0.09 (−0.25, 0.07)
Race—Non-Hispanic white (ref: other)	−0.01 (−0.18, 0.15)
Marital status—married (ref: other)	−0.18 (−0.33, −0.02)
Stage—stage II (ref: stage I)	0.15 (−0.02, 0.31)
Size (mm)	0.15 (0.07, 0.22)
Elixhauser comorbidity score	0.19 (0.11, 0.26)
Hepatitis B (Yes vs. ref: No)	−0.33 (−0.57, −0.10)
Hepatitis C	−0.03 (−0.20, 0.13)
Other or unspecified viral hepatitis	0.26 (−0.02, 0.54)
Nonviral hepatitis	−0.19 (−0.39, 0.00)
Alcoholic liver disease	0.13 (−0.07, 0.32)
Nonalcoholic cirrhosis	0.09 (−0.13, 0.31)
Portal hypertension	0.65 (0.48, 0.82)
Hepatic encephalopathy/coma	0.43 (0.19, 0.66)

## Data Availability

Data are available upon obtaining approval from NCI; for details, please see https://healthcaredelivery.cancer.gov/seermedicare/obtain/ (accessed on 20 April 2024).

## Supplementary Materials

The following supporting information can be downloaded at https://www.mdpi.com/article/10.3390/life14060661/s1: Figure S1: Distribution of estimated propensity scores (upper) and IPT weights (bottom) based on the samples thinned at every 250th iterations for visibility; Figure S2: Absolute standardized mean difference (SMD) before and after weighting; Table S1: Liver cancer treatment options summarized by NIH National Cancer Institute; Table S2: Diagnosis and procedure codes used to identify surgical procedures; Table S3: Diagnosis codes used to identify liver conditions; Table S4: Summary of treatment effects from 17 selected publications; Table S5: Results from the IPT weighted frequentist Weibull AFT model with covariates; Table S6: Results from the Bayesian Logistic Regression model for estimating propensity scores; Table S7: Results from the IPT weighted Bayesian Weibull AFT model with covariates [50,51,52].
