# Supplementary material for: Partial Hepatectomy and Ablation for Survival of Early-Stage Hepatocellular Carcinoma Patients: A Bayesian Emulation Analysis
Source: Life (Basel). 2024 May 22;14(6):661. doi: 10.3390/life14060661 (PMC11204969; doi:10.3390/life14060661)
Supplement: Supplementary file 1 [file life-14-00661-s001.zip › life-2998808-supplementary.pdf]

Supplementary Materials for "Partial hepatectomy and ablation for survival of early-stage hepatocellular carcinoma patients: a Bayesian emulation analysis"

1. Procedures for assembling cohort and defining treatments

1.1. Eligibility criteria

1. Primary first or only diagnosis of hepatocellular carcinoma (HCC) between 1/1/2007 and 12/31/2017, not diagnosed at autopsy or death, and not from NY, MA, and ID.

1) SEER site code for Hepatoma: 21071, and not from NY (62), MA (63), and ID (61) because of missing cancer-specific information.

2) HCC is heterogeneous with multiple pathological subtypes, mainly including fibrolamellar (ICD-O-3 code: 8171), scirrhous (8172), clear cell (8174), and sarcomatoid (spindle cell and pleomorphic) carcinoma (8173, 8175). The morphology codes also include HCC, NOS (8170).

2. HCC Stage I-II and tumor size less than or equal to 5 cm.

1) We used AJCC 6th for 2007-2015 and SEER combined stage for 2016-2017. The stage codes for derived AJCC 6th ed. T, N, M, and Stage variables include 10-19 (Stage I), 30-34 (Stage II); and 1A, 1B (Stage I), 2, 2A, 2B (Stage II) for derived SEER combined T, N, and M variables.

2) We used CS TUMOR SIZE_2004_2015 (codes: 1-50 & 991-995) for 2007-2015 and TUMOR SIZE SUMMARY_2016 for 2016-2017 (codes: 1-50);

3. Age 66-100 at diagnosis (we removed records with missing month of diagnosis information).

4. Remove records with conflicting death (in particular, died before diagnosis) or sex information.

5. Eligible patients had to have continuous enrollment in Medicare Part A (inpatient care) and Part B (outpatient services) with no health maintenance organization (HMO) enrollment. The enrollment should last from one year before diagnosis and until one year after diagnosis. We excluded patients with inadequate Medicare Part A and B enrollment or enrolled in HMO because claims were not all available.

6. Patients had either liver ablation or partial hepatectomy (resection or lobectomy) as the primary treatment recorded in either MedPAR and NCH or Outpatient Medicare records within one year after diagnosis.

1.2. Treatment identification

Based on the NIH liver cancer treatment guidelines, treatments options include the following.

Table S1. Liver cancer treatment options summarized by NIH National Cancer Institute.

**Stage**	**Treatment**	**Details**	**Notes**
Localized HCC	Total hepatectomy	With transplant. If a patient needs to wait for a donated liver, other treatments may be given.	Not of interest
	Partial hepatectomy	Wedge or segmental resection	We studied ablation vs. partial hepatectomy
		Lobectomy	Counted in analysis
		Extended lobectomy	Counted in analysis
	Ablation	Heat-Radio-frequency ablation (RFA)	Counted in analysis
		Alcohol (Percutaneous Ethanol Injection-PEI)	Counted in analysis
		Cryoablation	Counted in analysis
		Electroporation therapy	Counted in analysis
		Microwave therapy	Counted in analysis

		Laser thermal ablation	Counted in analysis
		High-intensity-focused-ultrasound (HIFU) ablation	Counted in analysis
Locally advanced or metastatic HCC	Embolization therapy	Transarterial embolization (TAE)	Not of interest
		Transarterial chemoembolization (TACE)	Not of interest
	Radiation therapy	Conformal radiation therapy	Not of interest
		Stereotactic body radiation therapy	Not of interest
		Proton beam radiation therapy	Not of interest
	Radiation therapy	For those ineligible for standard surgical resection	Not of interest
	Targeted therapy with sorafenib, lenvatinib, regorafenib, cabozantinib, bevacizumab, or ramucirumab		Not of interest
	Immune checkpoint inhibitor therapy with pembrolizumab, nivolumab with ipilimumab, or atezolizumab with the targeted therapy bevacizumab		Not of interest

Among those options, we studied the comparative effectiveness of ablation vs. partial hepatectomy in overall survival. The diagnosis and procedure codes used to identify specific surgeries are listed below.

Table S2. Diagnosis and procedure codes used to identify surgical procedures.

**Procedure**	**Codes**[1]
**Partial hepatectomy -** Wedge or segmental resection	**ICD-9 Procedure**: 50.22<br>**ICD-10 Procedure**:0FB00ZZ, 0FB03ZZ, 0FB04ZZ (Excision of Liver), 0FB10ZZ, 0FB13ZZ, 0FB14ZZ, 0FB20ZZ, 0FB23ZZ, 0FB24ZZ (Excision of Left/Right Lobe Liver)<br>**HCPCS**: 47120
**Partial hepatectomy –** Lobectomy and Extended lobectomy	**ICD-9 Procedure**: 50.3<br>**ICD-10 Procedure**: 0FT10ZZ, 0FT14ZZ, 0FT20ZZ, 0FT24ZZ (resection of left/right lobe liver)<br>**HCPCS**: 47122, 47125, 47130
**Ablation** - Heat-Radio-frequency ablation (RFA)	**ICD-9 Procedure**: 50.23, 50.24, 50.25, 50.26 (ablation in general)<br>**HCPCS**: 47370, 47380, 47382
**Ablation -** Alcohol (Percutaneous Ethanol Injection-PEI)	**ICD-9 Procedure**: 50.23, 50.24, 50.25, 50.26 (ablation in general)
**Ablation -** Cryoablation	**ICD-9 Procedure**: 50.23, 50.24, 50.25, 50.26 (ablation in general)<br>**HCPCS**: 47381, 47371, 47383
**Ablation -** Electroporation therapy	**ICD-9 Procedure**: 50.23, 50.24, 50.25, 50.26 (ablation in general)<br>**ICD-10 Procedure**: 0F510ZF, 0F513ZF, 0F514ZF, 0F520ZF, 0F523ZF, 0F524ZF
**Ablation -** Microwave therapy	**ICD-9 Procedure**: 50.23, 50.24, 50.25, 50.26 (ablation in general)
**Ablation -** Laser thermal ablation	**ICD-9 Procedure**: 50.23, 50.24, 50.25, 50.26 (ablation in general)
**Ablation -** High-intensity-focused-ultrasound (HIFU) ablation	**ICD-9 Procedure**: 50.23, 50.24, 50.25, 50.26 (ablation in general)<br>**HCPCS**: 0686T
[1]We searched the MedPAR, Outpatient, and NCH claims for all codes except where otherwise noted. All ICD codes have periods removed consistent with how they are recorded in the Medicare claims.	

1.3. Comorbidities: Elixhauser index and Liver diseases

Medicare MedPAR, Outpatient, and NCH were used to identify comorbidities. We used a baseline of one year to define comorbidity scores. Comorbidity was identified as when a patient had at least one diagnosis from the MedPAR file or at least two distinct diagnoses recorded >30 days apart from the Outpatient or NCH file in the 365 days before cancer diagnosis. The algorithm for the modified Elixhauser score was based on van Walraven (2009) [36], and ICD-9-CM and ICD-10-CM diagnosis codes of the Elixhauser conditions were obtained from Quan (2005) [50]. In addition, ICD-9-CM diagnosis codes that could indicate liver disease were individually tabulated in Table S3.

Table S3. Diagnosis codes used to identify liver conditions.

Conditions	ICD-9-CM Diagnosis[1]	ICD-10-CM Diagnosis[1]
**hepatitis B**	070.2, 070.20, 070.21, 070.22, 070.23, 070.3, 070.30, 070.31, 070.32, 070.33, V02.61	B16.0, B16.1, B16.2, B16.9, B18.0, B18.1, B19.10, B19.11, Z22.51
**hepatitis C**	070.41, 070.44, 070.51, 070.54, 070.7, 070.70, 070.71, V02.62	B17.10, B17.11, B18.2, B19.20, B19.21, Z22.52
**other viral hepatitis (A,D,E, or unspecified)**	070.0, 070.1, 070.21, 070.23, 070.31, 070.33, 070.42, 070.43, 070.52, 070.53, 070.59, 070.6, 070.9, V02.6, V02.60, V02.69	B15.0, B15.9, B17.0, B17.2, B17.8, B17.9, B18.8, B18.9, B19.0, B19.9, Z22.50, Z22.59
**nonviral hepatitis**	571.40, 571.41, 571.42, 571.49, 573.3	K71, K71.0, K71.1, K71.10, K71.11, K71.2, K71.3, K71.4, K71.5, K71.50, K71.51, K71.6, K71.7, K71.8, K71.9, K73, K73.0, K73.1, K73.2, K73.8, K73.9, K75.2, K75.3, K75.4, K75.81
**alcoholic liver disease**	571.0, 571.1, 571.2, 571.3	K70, K70.0, K70.1, K70.10, K70.11, K70.2, K70.3, K70.30, K70.31, K70.4, K70.40, K70.41, K70.9
**nonalcoholic cirrhosis**	571.5, 571.6, 571.8, 571.9	K74, K74.0, K74.00, K74.01, K74.02, K74.1, K74.2, K74.3, K74.4, K74.5, K74.6, K74.60, K74.69
**portal hypertension**	572.3; ascites (789.5, 789.51, 789.59); esophageal varices (456.0, 456.1, 456.2, 456.20, 456.21); hepatorenal syndrome (572.4)	K76.6; ascites (R18, R18.0, R18.8); esophageal varices (I85, I85.0, I85.00, I85.01, I85.1, I85.10, I85.11); hepatorenal syndrome (K76.7)
**hepatic encephalopathy/coma**	070.4, 070.41, 070.42, 070.43, 070.44, 070.49, 070.6, 572.2	B17.11, B19.0, B19.11, B19.21

[1]*We searched the MedPAR, Outpatient, and NCH claims for all codes except where otherwise noted. All ICD codes have periods removed consistent with how they are recorded in the Medicare claims*

2. Prior information from the published literature

2.1. List of publications that contain relevant treatment effect information

1. Ko SE, Lee MW, Ahn S, Rhim H, Kang TW, Song KD, Kim JM, Choi GS, Cha DI, Min JH, Sinn DH, Choi MS, Lim HK. Laparoscopic Hepatic Resection Versus Laparoscopic Radiofrequency Ablation for Subcapsular Hepatocellular Carcinomas Smaller Than 3 cm: Analysis of Treatment Outcomes Using Propensity Score Matching. Korean J Radiol. 2022 Jun;23(6):615-624.
2. Hur MH, Lee JH, Kim JY, Hong JH, Park MK, Cho HJ, Choi NR, Kim J, Kim MA, Nam JY, Lee YB, Cho EJ, Yu SJ, Kim YJ, Lee DH, Lee JM, Hong SK, Yi NJ, Lee KW, Suh KS, Yoon JH. Comparison of Overall Survival between Surgical Resection and Radiofrequency Ablation for Hepatitis B-Related Hepatocellular Carcinoma. Cancers (Basel). 2021 Nov 29;13(23):6009.
3. Lee J, Jin YJ, Shin SK, Kwon JH, Kim SG, Suh YJ, Jeong Y, Yu JH, Lee JW, Kwon OS, Nahm SW, Kim YS. Surgery versus radiofrequency ablation in patients with Child- Pugh class-A/single small (≤3 cm) hepatocellular carcinoma. Clin Mol Hepatol. 2022 Apr;28(2):207-218. 3
4. Li YC, Chen PH, Yeh JH, Hsiao P, Lo GH, Tan T, Cheng PN, Lin HY, Chen YS, Hsieh KC, Hsieh PM, Lin CW. Clinical outcomes of surgical resection versus radiofrequency ablation in very-early-stage hepatocellular carcinoma: a propensity score matching analysis. BMC Gastroenterol. 2021 Nov 8;21(1):418.
5. Chen S, Ma W, Shen L, Wu Y, Qi H, Cao F, Huang T, Fan W. Recurrence Beyond the Milan Criteria of HBV-Related Single Hepatocellular Carcinoma of 2-3 cm: Comparison of Resection and Ablation. Front Oncol. 2021 Oct 18;11:757149.
6. Xie W, Tan J, Li B, Chen S, Liu B, Shen J, Fu S, Kuang M, Sun K, Zeng X. Comparison of Hepatic Resection with Percutaneous Ablation for Hepatocellular Carcinoma in the Caudate Lobe Within Milan Criteria. J Gastrointest Surg. 2022 Feb;26(2):323-332.
7. Bai S, Yang P, Xie Z, Li J, Lei Z, Xia Y, Qian G, Zhang B, Pawlik TM, Lau WY, Shen F. Preoperative Estimated Risk of Microvascular Invasion is Associated with Prognostic Differences Following Liver Resection Versus Radiofrequency Ablation for Early Hepatitis

B Virus-Related Hepatocellular Carcinoma. Ann Surg Oncol. 2021 Dec;28(13):8174-8185.

8. Matsumoto M, Yanaga K, Shiba H, Wakiyama S, Sakamoto T, Futagawa Y, Gocho T, Ishida Y, Ikegami T. Treatment of intrahepatic recurrence after hepatectomy for hepatocellular carcinoma. Ann Gastroenterol Surg. 2021 Feb 24;5(4):538-552.

9. Chen ZY, Guo ZX, Lu LH, Mei J, Lin WP, Li SH, Wei W, Guo RP. The predictive value of vessels encapsulating tumor clusters in treatment optimization for recurrent early-stage hepatocellular carcinoma. Cancer Med. 2021 Aug;10(16):5466-5474.

10. Conticchio M, Inchingolo R, Delvecchio A, Laera L, Ratti F, Gelli M, Anelli F, Laurent A, Vitali G, Magistri P, Assirati G, Felli E, Wakabayashi T, Pessaux P, Piardi T, di Benedetto F, de'Angelis N, Briceño J, Rampoldi A, Adam R, Cherqui D, Aldrighetti LA, Memeo R. Radiofrequency ablation vs. surgical resection in elderly patients with hepatocellular carcinoma in Milan criteria. World J Gastroenterol. 2021 May 14;27(18):2205-2218.

11. Chua DW, Koh YX, Syn NL, Chuan TY, Yao TJ, Lee SY, Goh BKP, Cheow PC, Chung AY, Chan CY. Repeat hepatectomy versus radiofrequency ablation in management of recurrent hepatocellular carcinoma: an average treatment effect analysis. Ann Surg Oncol. 2021 Nov;28(12):7731-7740.

12. Rho SY, Lee HW, Kim DY, Kim KS. Current Status of Therapeutic Choice and Feasibility for Patients with Hepatocellular Carcinoma Aged ≥ 70 Years: A Nationwide Cancer Registry Analysis. J Hepatocell Carcinoma. 2021 Apr 30;8:321-332.

13. Santambrogio R, Barabino M, D'Alessandro V, Iacob G, Opocher E, Gemma M, Zappa MA. Micronvasive behaviour of single small hepatocellular carcinoma: which treatment? Updates Surg. 2021 Aug;73(4):1359-1369.

14. Wu CC, Tseng CW, Tseng KC, Chen YC, Wu TW, Chang SY, Chang YJ, Chao YC, Hsu CS. Radiofrequency ablation versus surgical resection for the treatment of solitary hepatocellular carcinoma 2 cm or smaller: A cohort study in Taiwan. J Formos Med Assoc. 2021 May;120(5):1249-1258.

15. Oh JH, Sinn DH, Choi GS, Kim JM, Joh JW, Kang TW, Hyun D, Kang W, Gwak GY, Paik YH, Lee JH, Koh KC, Paik SW, Choi MS. Comparison of outcome between liver resection, radiofrequency ablation, and transarterial therapy for multiple small hepatocellular carcinoma within the Milan criteria. Ann Surg Treat Res. 2020 Oct;99(4):238-246.

16. Yang JD, Luu M, Singal AG, Noureddin M, Kuo A, Ayoub WS, Sundaram V, Kotler H, Kim IK, Todo T, Voidonikolas G, Brennan TV, Kosari K, Klein AS, Hendifar A, Lu SC, Nissen NN, Gong J. Factors Associated With Detection and Survival of T1 Hepatocellular Carcinoma in the United States: National Cancer Database Analysis. J Natl Compr Canc Netw. 2020 Sep;18(9):1210-1220.

17. Lee HA, Lee YS, Kim BK, Jung YK, Kim SU, Park JY, Kim JH, An H, Kim DY, Yim HJ, Ahn SH, Yeon JE, Byun KS, Han KH, Um SH, Seo YS. Change in the Recurrence Pattern and Predictors over Time after Complete Cure of Hepatocellular Carcinoma. Gut Liver. 2021 May 15;15(3):420-429.

2.2. Prior information on treatment effect contained in the above publications

We have summarized the estimated hazard ratios with 95% confidence interval of the 17 selected publications in the following Table S4.

Table S4. Summary of treatment effects from 17 selected publications.

Reference	HR	95% CI	P value
**Ko et al. (2022)**	1.330	(0.120, 1.540)	0.818
**Hur et al. (2021)**	4.226	(1.938, 9.215)	<0.001
**Lee et al. (2022)**	0.698	(0.396, 1.232)	0.215
**Li et al. (2021)**	1.750	(1.080, 3.110)	0.046
**Chen et al. (2021)**	4.226	(1.938, 9.215)	<0.001
**Xie et al. (2022)**	5.970	(1.480, 24.120)	0.012
**Bai et al. (2021)**	2.128	(1.190, 3.846)	0.011

**Matsumoto et al. (2021)**	2.857	(0.772, 10.576)	0.116
	1.631	(0.361, 7.368)	0.525
**Chen et al. (2021)**	1.133	(0.815, 1.575)	0.459
**Conticchio (2021)**	1.460	(1.100, 1.790)	0.001
**Chua et al. (2021)**	1.120	(0.730, 1.730)	0.601
**Rho et al. (2021)**	2.830	(1.411, 5.677)	0.003
	1.593	(1.067, 2.379)	0.023
**Santambrogio et al. (2021)**	1.577	(1.069, 2.326)	0.021
**Wu et al. (2021)**	1.605	(1.101, 2.341)	0.014
**Oh et al. (2020)**	2.271	(1.427, 3.616)	0.001
**Yang et al. (2020)**	1.497	(1.075, 2.083)	0.017
**Lee et al. (2021)**	1.848	(1.541, 2.216)	<0.001

3. Bayesian propensity score analysis results

3.1. Propensity scores and weights

To further visualize the propensity score analysis results, we showed in Figure S1 the distributions of propensity scores and the corresponding weights across 20 different MCMC iterations.

Figure S1. Distribution of estimated propensity scores (upper) and IPT weights (bottom) based on the samples thinned at every 250th iterations for visibility.

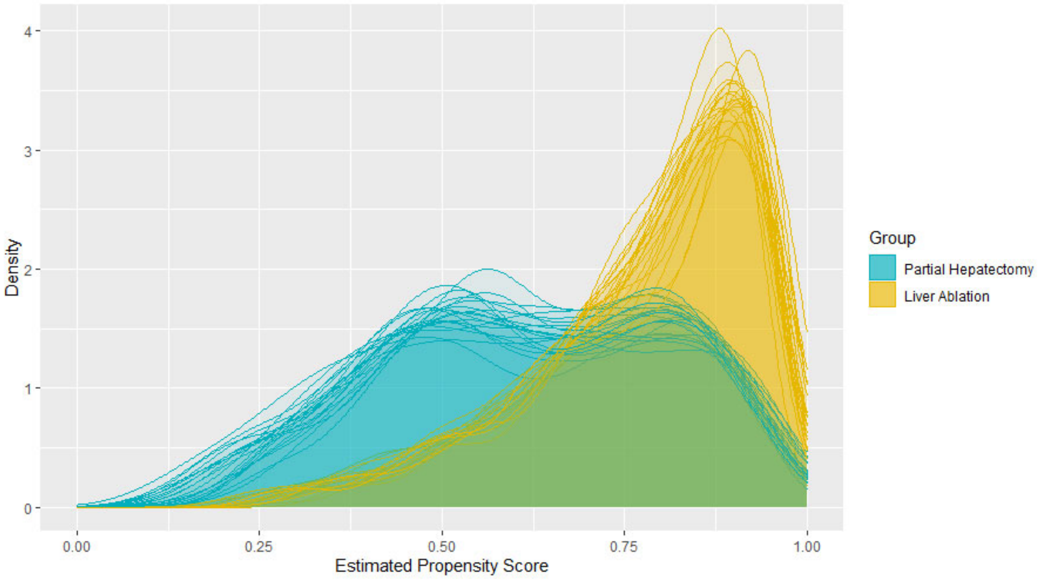

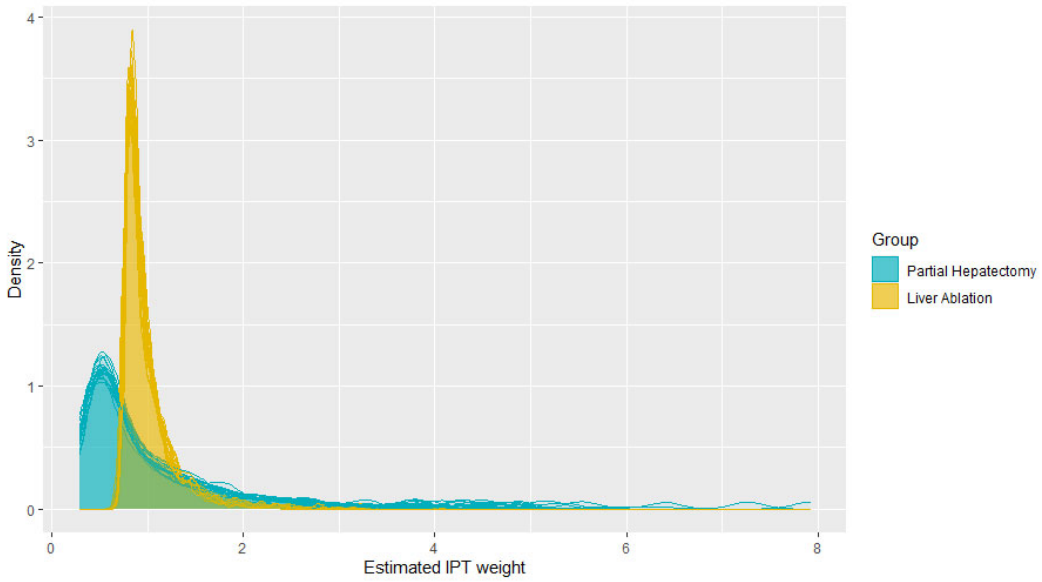

3.2. Standard mean differences

The visualization of the absolute standardized mean difference (SMD) results was presented in Figure S2. We displayed the distribution of the SMD values for each variable (colored in red) and the exact SMD value for each variable before weighting (denoted as blue points). Prior to weighting, the SMD values for hepatitis C, hepatitis B, portal hypertension, nonviral hepatitis, alcoholic liver disease, nonalcoholic cirrhosis, hepatic encephalopathy/coma, and tumor size were above 0.1. The distributions of SMD values showed that most of the SMD values significantly decreased to be below or close to 0.1 with weighting, with which the two treatment groups were considered balanced [51,52].

Figure S2. Absolute standardized mean difference (SMD) before and after weighting.

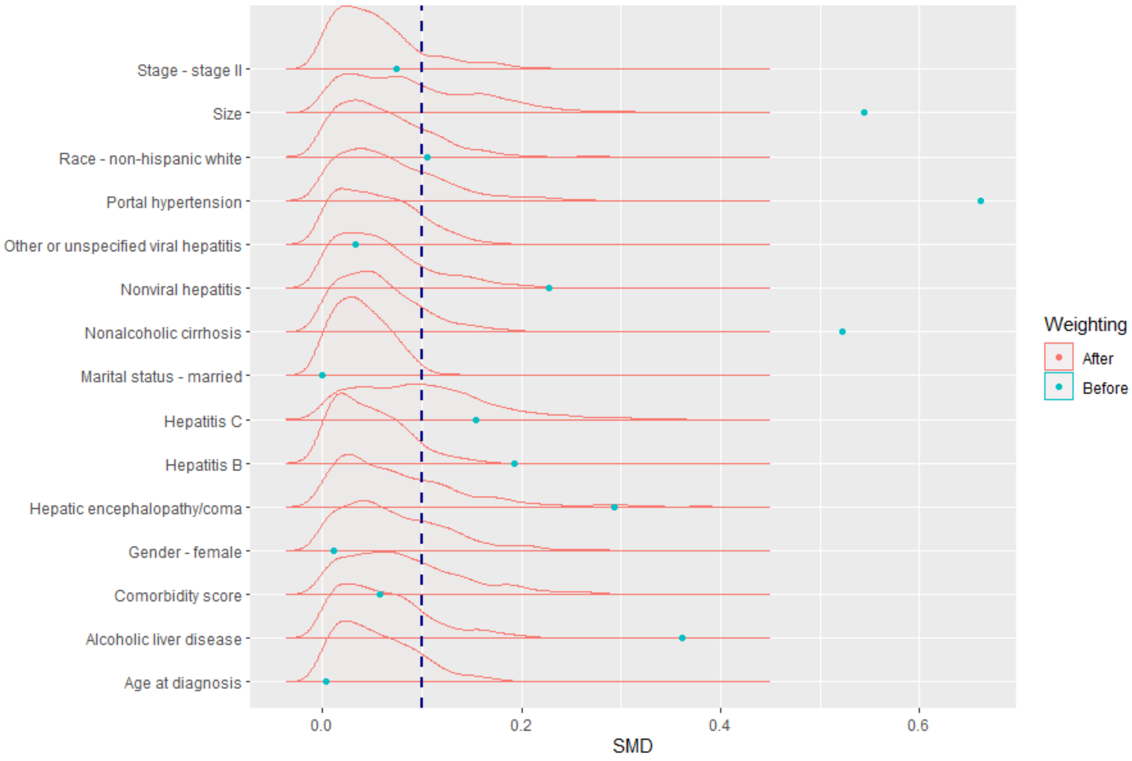

4. Frequentist analysis results

We also conducted the frequentist analysis. We utilized a propensity score analysis and modeled treatment assignment as a function of the baseline characteristics using logistic regression and then constructed the outcome model incorporating the propensity scores via inverse probability treatment (IPT) weighting. To be cautious, as in real RCT analysis, we also included confounders in the outcome model. Supplementary Table 5 contains the adjusted log hazard ratio and 95% confidence interval for each variable using the standard frequentist approach. Ablation was observed to have a significant association with survival, with an estimated log HR of 0.27 (HR = 1.31) and 95% confidence interval (0.06, 0.47) – the corresponding 95% confidence interval for HR is (1.06, 1.60). Other variables significantly associated with survival were age at diagnosis, marital status, tumor size, Elixhauser comorbidity score, Hepatitis B, Portal hypertension, and Hepatic encephalopathy/coma. One limitation of this analysis is that valuable prior information cannot be accommodated. Additionally, in the outcome modeling, the propensity scores are treated as fixed, and the uncertainty associated with estimating those scores is not accounted for.

Table S5. Results from the IPT weighted frequentist Weibull AFT model with covariates.

Variable	Log hazard ratio (95% confidence interval)
Surgery – ablation (ref: partial hepatectomy)	0.27 (0.06, 0.47)
Age at diagnosis	0.21 (0.14, 0.30)
Sex – female (ref: male)	-0.08 (-0.23, 0.10)
Race – Non-Hispanic white (ref: other)	-0.01 (-0.20, 0.15)
Marital status – married (ref: other)	-0.16 (-0.31, -0.01)
Stage – stage II (ref: stage I)	0.13 (-0.03, 0.29)
Size (mm)	0.13 (0.05, 0.22)
Elixhauser comorbidity score	0.17 (0.09, 0.25)
Hepatitis B (Yes vs. ref: No)	-0.28 (-0.52, -0.06)
Hepatitis C	-0.03 (-0.19, 0.14)
Other or unspecified viral hepatitis	0.24 (-0.03, 0.48)
Nonviral hepatitis	-0.16 (-0.37, 0.04)
Alcoholic liver disease	0.11 (-0.10, 0.35)
Nonalcoholic cirrhosis	0.07 (-0.12, 0.28)
Portal hypertension	0.57 (0.41, 0.77)
Hepatic encephalopathy/coma	0.38 (0.11, 0.72)

5. Subgroup analysis

Table S6. Results from the Bayesian Logistic Regression model for estimating propensity scores.

Variable	Log hazard ratio (95% credible interval)
Age at diagnosis	0.22 (0.01, 0.44)
Sex – female (ref: male)	-0.33 (-0.74, 0.11)
Race – Non-Hispanic white (ref: other)	0.17 (-0.31, 0.66)
Marital status – married (ref: other)[1]	-0.17 (-0.60, 0.31)
Stage – stage II (ref: stage I)	0.27 (-0.22, 0.82)
Size (mm)	0.00 (-0.27, 0.20)
Elixhauser comorbidity score	-0.23 (-0.46, -0.02)
Hepatitis B (Yes vs. ref: No)	0.20 (-0.45, 0.88)
Hepatitis C	0.15 (-0.29, 0.68)
Other or unspecified viral hepatitis	-0.04 (-0.78, 0.74)
Nonviral hepatitis	0.33 (-0.25, 0.99)
Alcoholic liver disease	0.84 (0.11, 1.56)
Nonalcoholic cirrhosis	0.61 (0.02, 1.18)
Portal hypertension	0.86 (0.41, 1.31)
Hepatic encephalopathy/coma	0.93 (-0.04, 2.08)

[1]For the subgroup analysis, marital status was no longer balanced between the two arms and thus included in the propensity score model.

Table S7. Results from the IPT weighted Bayesian Weibull AFT model with covariates.

Variable	Log hazard ratio (95% credible interval)
Surgery – ablation (ref: partial hepatectomy)	0.14 (-0.13, 0.42)
Age at diagnosis	0.25 (0.14, 0.35)
Sex – female (ref: male)	0.00 (-0.23, 0.22)
Race – Non-Hispanic white (ref: other)	-0.09 (-0.32 0.13)
Marital status – married (ref: other)	-0.23 (-0.44, -0.02)
Stage – stage II (ref: stage I)	0.21 (-0.02, 0.44)
Size (mm)	0.10 (-0.01, 0.21)
Elixhauser comorbidity score	0.32 (0.22, 0.42)
Hepatitis B (Yes vs. ref: No)	-0.41 (-0.71, -0.11)
Hepatitis C	0.03 (-0.19, 0.25)
Other or unspecified viral hepatitis	0.19 (-0.18, 0.54)
Nonviral hepatitis	0.02 (-0.23, 0.26)
Alcoholic liver disease	0.21 (-0.07, 0.49)
Nonalcoholic cirrhosis	-0.05 (-0.38, 0.31)
Portal hypertension	0.53 (0.30, 0.76)
Hepatic encephalopathy/coma	0.57 (0.25, 0.89)
